# Exploratory analysis of machine learning models for state and trait anxiety based on Spielberger questionnaire data in nursing students

**DOI:** 10.1186/s12909-026-08842-3

**Published:** 2026-02-21

**Authors:** Reza Salehinia, Sajjad Salehian, Marzieh Nasiri Sangari, Mohammad Amin Nasiri Sangari, Hossein Abbassian

**Affiliations:** 1 Department of Surgical Technology, Tabas School of Nursing, Birjand University of Medical Sciences, Birjand, Iran; 2 Department of Nursing, Tabas School of Nursing, Birjand University of Medical Sciences, Birjand, Iran; 3 Department of Mechanical Engineering, School of Mechanical Engineering, Shiraz University, Shiraz, Iran; 4 Tabas School of Nursing, Birjand University of Medical Sciences, Birjand, Iran

**Keywords:** Machine learning, Anxiety, State-Trait anxiety, Nursing students

## Abstract

**Background and objective:**

This study aimed to explore the ability of machine learning models to assess state and trait anxiety using data collected from the Spielberger State-Trait Anxiety Inventory (STAI). Considering the significant impact of mental health on the academic and professional performance of medical students, the research sought to determine whether machine learning could complement traditional assessment methods and provide insights into the relative influence of demographic and physiological factors on anxiety levels.

**Methods:**

A census sampling approach was applied, including all 106 eligible students from the Tabas Faculty of Nursing. Participants with a history of anxiety disorders or use of psychoactive medications were excluded. State and trait anxiety were measured using the STAI. Data analysis was performed using SPSS and MATLAB. Bivariate tests (Kruskal-Wallis and Chi-Square) examined associations between anxiety and demographic/physiological variables. Multiple linear regression was used as an exploratory modeling approach to predict anxiety, with model performance evaluated via 10-fold cross-validation, RMSE, and R². Standardized coefficients were calculated to estimate the relative importance of predictors.

**Findings:**

Participants had a mean age of 21.36 years, with 58.5% being female. Most demographic and physiological variables were not significantly associated with anxiety, except for marital status and the strong correlation between state and trait anxiety (*p* < .001). The regression model captured overall trends in anxiety scores but showed moderate predictive accuracy, particularly for extreme values (state anxiety: RMSE ≈ 8.89, R² ≈ 0.13; trait anxiety: RMSE ≈ 8.70, R² ≈ 0.11). Gender, academic major, and some physiological factors (e.g., SpO₂, body temperature) were the most influential predictors, while other variables had minimal contribution. These results reflect relative associations rather than precise individual-level predictions.

**Conclusion:**

This study confirms the strong relationship between state and trait anxiety and highlights the relative influence of social and demographic factors over physiological indicators in this population. Although predictive accuracy was moderate, machine learning models can reveal complex patterns that may be overlooked by traditional statistical methods, offering guidance for exploratory assessment and targeted interventions for anxiety among nursing students.

**Supplementary Information:**

The online version contains supplementary material available at 10.1186/s12909-026-08842-3.

## Introduction

Anxiety is recognized as one of the most prevalent psychological issues, significantly affecting mental health, individual performance, and quality of life [1]. This concern is particularly critical among medical students, who are frequently exposed to stressful clinical environments, heavy academic workloads, and pressures associated with preparing for their professional careers. Consequently, they are at an elevated risk of experiencing high levels of anxiety [2]. Such anxiety can adversely impact academic performance, clinical decision-making, and overall well-being [3]. Anxiety manifests in distinct forms, including state anxiety, which is transient and situation-dependent, and trait anxiety, considered a stable personality characteristic [4]. Traditional assessment tools, such as the Spielberger State-Trait Anxiety Inventory (STAI), are widely recognized for their validity and efficiency; however, they may be limited in detecting complex patterns or situational variations in anxiety [5]. These limitations highlight the potential utility of advanced analytical methods for psychological data. In this context, machine learning has emerged as a powerful approach for analyzing multidimensional and complex datasets. Machine learning algorithms can identify patterns in physiological and demographic data, such as heart rate variability, blood oxygen levels, and blood pressure, which are often noisy and variable, and extract meaningful insights [6]. Feature identification methods, including support vector machines and neural networks, can highlight key predictors of anxiety and aid in designing personalized interventions or early warning systems for mental health management [7–9]. Despite these advantages, the accuracy and utility of machine learning models depend critically on data quality, model design, and proper interpretation of results [10]. Given these developments, machine learning has recently gained attention as a complementary tool for psychological data analysis. By analyzing data derived from standardized questionnaires, these approaches can uncover patterns that may be challenging to detect with traditional methods. However, the performance of machine learning models relative to conventional tools such as the STAI remains underexplored. Most previous studies have focused either on psychological questionnaires or on machine learning independently, without integrating both approaches. This study aims to investigate the ability of machine learning models to predict both state and trait anxiety using data collected from the STAI among students of the Tabas Faculty of Nursing. The primary objective is to determine whether machine learning can provide meaningful insights into the relative influence of demographic and physiological variables on anxiety levels, complementing traditional assessment methods and supporting more informed strategies for intervention and prevention.

## Materials and methods

A census sampling method was applied, including all eligible students enrolled at the Tabas Faculty of Nursing (*N* = 106). Inclusion criteria required informed consent and willingness to participate. To minimize bias and improve result accuracy, exclusion criteria included: (1) a history or diagnosis of any anxiety disorder or other mental illness, and (2) use of tranquilizers or psychoactive medications within the past six months. This approach ensured that the data reflected the anxiety status of a general and relatively healthy student population. Anxiety levels were assessed using the State-Trait Anxiety Inventory (STAI), a widely used 40-item self-report questionnaire that measures both state anxiety (current feelings) and trait anxiety (a stable personality characteristic). The STAI was originally developed by Spielberger et al. [11, 12]. In the Iranian context, the reliability of STAI has been confirmed, with reported Cronbach’s alpha of 0.92 for the state anxiety scale and test-retest reliability of 0.65–0.86 for the trait anxiety scale in student populations [13]. To ensure sufficient statistical power, a post-hoc power analysis was conducted using G*Power software, considering a medium effect size (d = 0.3), significance level (α = 0.05), and power (1 − β = 0.95), confirming the sample size was adequate. Descriptive statistics were used to summarize demographic and physiological variables. Bivariate analyses examined associations between these variables and both state and trait anxiety. For non-normally distributed continuous variables, the Kruskal-Wallis test was applied, and for nominal data, the Chi-Square or Fisher’s Exact Test was used. Missing values were handled appropriately: variables with meaningful absence (e.g., physical activity, eye score) were coded as zero, and for other continuous variables, missing data were imputed using the median.The dataset was subsequently used for exploratory multiple linear regression modeling to examine the relative contributions of demographic and physiological variables to anxiety scores, with model performance evaluated using 10-fold cross-validation, R², and RMSE.

In the second phase of the study, multiple linear regression was applied as an exploratory approach to examine associations between demographic and physiological variables and state and trait anxiety scores. The primary aim was not to achieve highly accurate predictions, but rather to explore the relative contributions of each predictor and the proportion of variance in anxiety scores explained by these variables. Demographic and physiological data were obtained from an Excel file and included age, gender, academic major, height, weight, systolic and diastolic blood pressure, heart rate, peripheral oxygen saturation (SpO₂), respiratory rate, body temperature, physical activity, and eye score. Anxiety scores were obtained from a separate file and merged with the predictors. One irrelevant column was removed before analysis. Missing values were handled to reduce potential bias. For variables where the absence of data carried meaningful information (physical activity and eye score), missing values were coded as zero, indicating no recorded activity or absent optical correction. For other continuous variables, missing values were imputed using the median to preserve the distributional characteristics of the data. This approach is exploratory and may affect model precision. All continuous predictors were standardized using z-score normalization to ensure comparability and facilitate interpretation of regression coefficients. Rather than relying on a single train–test split, model performance was assessed using 10-fold cross-validation across the entire dataset. Each participant contributed to both training and validation sets, reducing sampling variability and mitigating overfitting. In each fold, the model was trained on k–1 subsets and tested on the remaining subset. Performance metrics included the coefficient of determination (R²) and root mean square error (RMSE), reported as mean ± standard deviation. For interpretative purposes, a final multiple linear regression model was fitted to the full standardized dataset. Standardized regression coefficients were extracted, and the relative importance of each predictor was calculated by scaling the absolute values of the coefficients relative to the largest coefficient. These normalized values provide a comparison of effect magnitudes but do not imply absolute or causal significance. All analyses were performed in MATLAB (R2023a, MathWorks, Natick, MA, USA). Given the moderate explained variance, limited sample size, and lack of external validation, the results should be interpreted as exploratory and hypothesis-generating rather than predictive for individual cases.

During the preparation of this manuscript, the authors used a large language model to enhance grammatical structure, ensure clarity in English, and refine the academic tone throughout the document. The authors have thoroughly reviewed, edited, and validated all outputs and take full responsibility for the accuracy, scientific content, and integrity of the final manuscript.

### Findings

The descriptive results for 106 students from the Tabas Faculty of Nursing show that the mean age of the sample was 21.36 ± 2.239 years. In terms of demographic composition, 58.5% of the participants were female (*n* = 62) and 41.5% were male (*n* = 44). The majority of the sample (92.5%) was single, while 7.5% (*n* = 8) were married. The students were distributed across three fields of study: Nursing (48.1%), Operating Room (33.0%), and Emergency Medical Services (18.9%). Table 1.

**Table 1:** Frequency distribution of demographic and clinical variables of participants

Variable	Category	Frequency (*N*)	Percentage (%)
Gender	Female	62	58.50%
	Male	44	41.50%
Marital Status	Single	98	92.50%
	Married	8	7.50%
Field of Study	Nursing	51	48.10%
	Operating Room	35	33.00%
	Emergency Medical Services	20	18.90%
Physical Activity	Yes	79	74.50%
	No	27	25.50%
BMI	Underweight	14	13.20%
	Normal	72	67.90%
	Overweight	16	15.10%
	Obese	4	3.80%
Use of Glasses	Yes	42	39.60%
	No	64	60.40%
State Anxiety	None	7	6.60%
	Mild	51	48.10%
	Moderate	32	30.20%
	Severe	16	15.10%
Trait Anxiety	None	25	23.60%
	Mild	46	43.40%
	Moderate	29	27.40%
	Severe	6	5.70%

Regarding lifestyle and physiological indicators, the mean body mass index (BMI) was 22.18 ± 3.54, with the majority of students (67.9%) falling within the normal range. Additionally, 74.5% of the sample reported physical activity. The results related to vital physiological indicators showed that the mean systolic and diastolic blood pressure were 115.13 ± 35.00 and 72.35 ± 20.93 mmHg, respectively. The average heart rate was 87.42 ± 15.98 beats per minute, and the average respiratory rate was 17.25 ± 9.38 breaths per minute. The mean blood oxygen saturation (SpO2) was 97.98 ± 1.92, and the mean body temperature was 36.63 ± 0.84, both within the normal range.

Results on anxiety status indicated that the mean scores for state anxiety and trait anxiety were 42.59 ± 9.58 and 41.52 ± 9.28, respectively. The majority of students reported mild to moderate levels of anxiety, with 48.1% having mild state anxiety and 43.4% having mild trait anxiety.

Based on the results of the present study, a significant relationship was not observed between state and trait anxiety and most demographic and physiological variables. In other words, variables such as age, gender, field of study, Body Mass Index (BMI), blood pressure, heart rate, SpO2​​, respiration rate, temperature, physical activity, and use of glasses were not independently associated with the level of state or trait anxiety in the students. However, the results revealed two significant correlations: the first finding was a significant association between marital status and trait anxiety (*p* = 0.019). The second and most important finding was a strong and significant relationship between state anxiety and trait anxiety (*p* < 0.001). This result indicates that these two aspects of anxiety are highly interconnected, meaning that as the level of one increases, the likelihood of an increase in the other also rises Table 2.

**Table 2:** Results of bivariate analysis of the relationship between anxiety and other variables

Dependent Variable	Type of Anxiety	Statistical Test	*p*-value
Age	State	Kruskal-Wallis	0.811
	Trait	Kruskal-Wallis	0.86
BMI	State	ANOVA	0.295
	Trait	ANOVA	0.64
Systolic Blood Pressure	State	Kruskal-Wallis	0.876
	Trait	Kruskal-Wallis	0.615
Diastolic Blood Pressure	State	Kruskal-Wallis	0.434
	Trait	Kruskal-Wallis	0.199
Heart Rate	State	Kruskal-Wallis	0.08
	Trait	Kruskal-Wallis	0.193
SpO2​​	State	Kruskal-Wallis	0.154
	Trait	Kruskal-Wallis	0.67
Respiration Rate	State	Kruskal-Wallis	0.78
	Trait	Kruskal-Wallis	0.06
Temperature	State	Kruskal-Wallis	0.38
	Trait	Kruskal-Wallis	0.47
Gender	State	Chi-Square	0.82
	Trait	Chi-Square	0.8
Marital Status	State	Fisher’s Exact Test	0.61
	Trait	Fisher’s Exact Test	0.019
Field of Study	State	Fisher’s Exact Test	0.84
	Trait	Fisher’s Exact Test	0.38
Physical Activity	State	Chi-Square	0.2
	Trait	Chi-Square	0.086
Use of Glasses	State	Chi-Square	0.67
	Trait	Chi-Square	0.21
State Anxiety	Trait	Fisher’s Exact Test	< 0.001

Logistic regression analysis showed that trait anxiety was not a significant predictor of marital status (*p* = 0.73). This means that, according to this model, the level of trait anxiety alone cannot predict marital status. However, the odds ratio (OR) of 0.862 indicates that for every one-unit increase in trait anxiety, the probability of being married decreases very slightly, a finding that is not statistically significant.

### Model performance for state anxiety

**Fig. 1:**
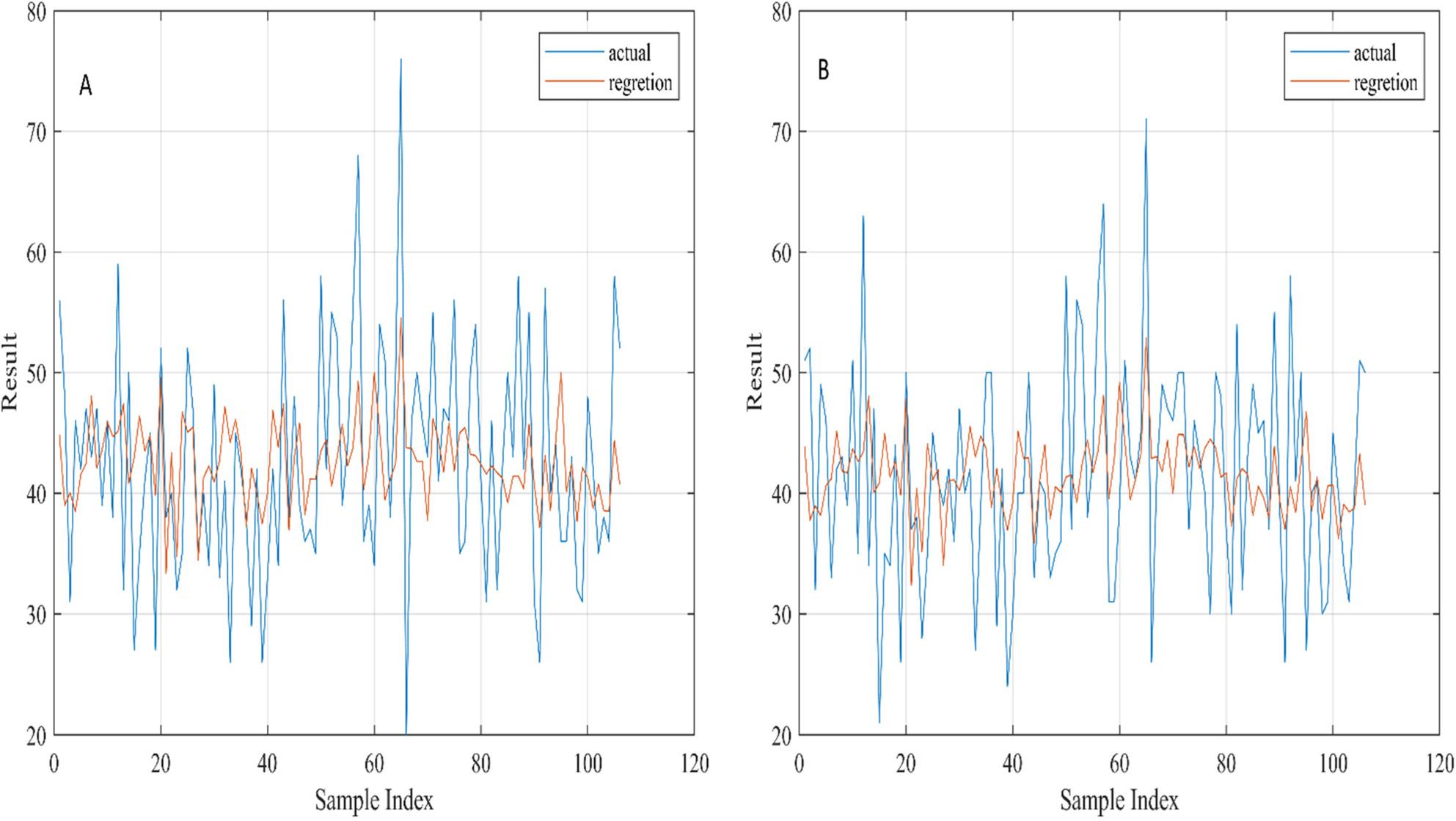
Exploratory visualization of actual and predicted anxiety scores. **A** Comparison of actual and predicted state anxiety scores across the entire dataset. **B** Comparison of actual and predicted trait anxiety scores across the entire dataset, illustrating the smoothing effect typical of linear regression predictions. Note: The scores in this chart are from the provided sample data table. While the regression model captures general trends in anxiety levels, predictions for extreme values show some smoothing. Model performance was formally assessed using 10-fold cross-validation. These plots are intended to show general trends rather than precise predictive accuracy for individual cases

The predictive performance of the multiple linear regression model for state anxiety is shown in Fig. 1, Plot A. This plot compares the actual anxiety scores (blue line) with predicted values (orange line) across the entire dataset. The model captures the general trend of the data but does not precisely follow extreme peaks and troughs, indicating moderate predictive capability. Predicted values are smoother and show less variance than the actual scores, which is typical of linear regression models that tend to underpredict high values and overpredict low values. Across 10-fold cross-validation, the model achieved an R² of approximately 0.22 ± 0.08 and an RMSE of 7.91 ± 0.42, reflecting moderate explanatory ability rather than high predictive accuracy.

### Model performance for trait anxiety

Plot B in Fig. 1 illustrates the model’s predictions for trait anxiety, derived from a separate column as described in the Methods. The model generally follows the trend of actual scores, with slightly better alignment in some sections compared to state anxiety. Deviations at extreme values remain, showing similar limitations in predictive accuracy. Cross-validated performance for trait anxiety resulted in an R² of 0.28 ± 0.09 and an RMSE of 7.76 ± 0.39, indicating moderate predictive capability.

### Feature importance

**Fig. 2:**
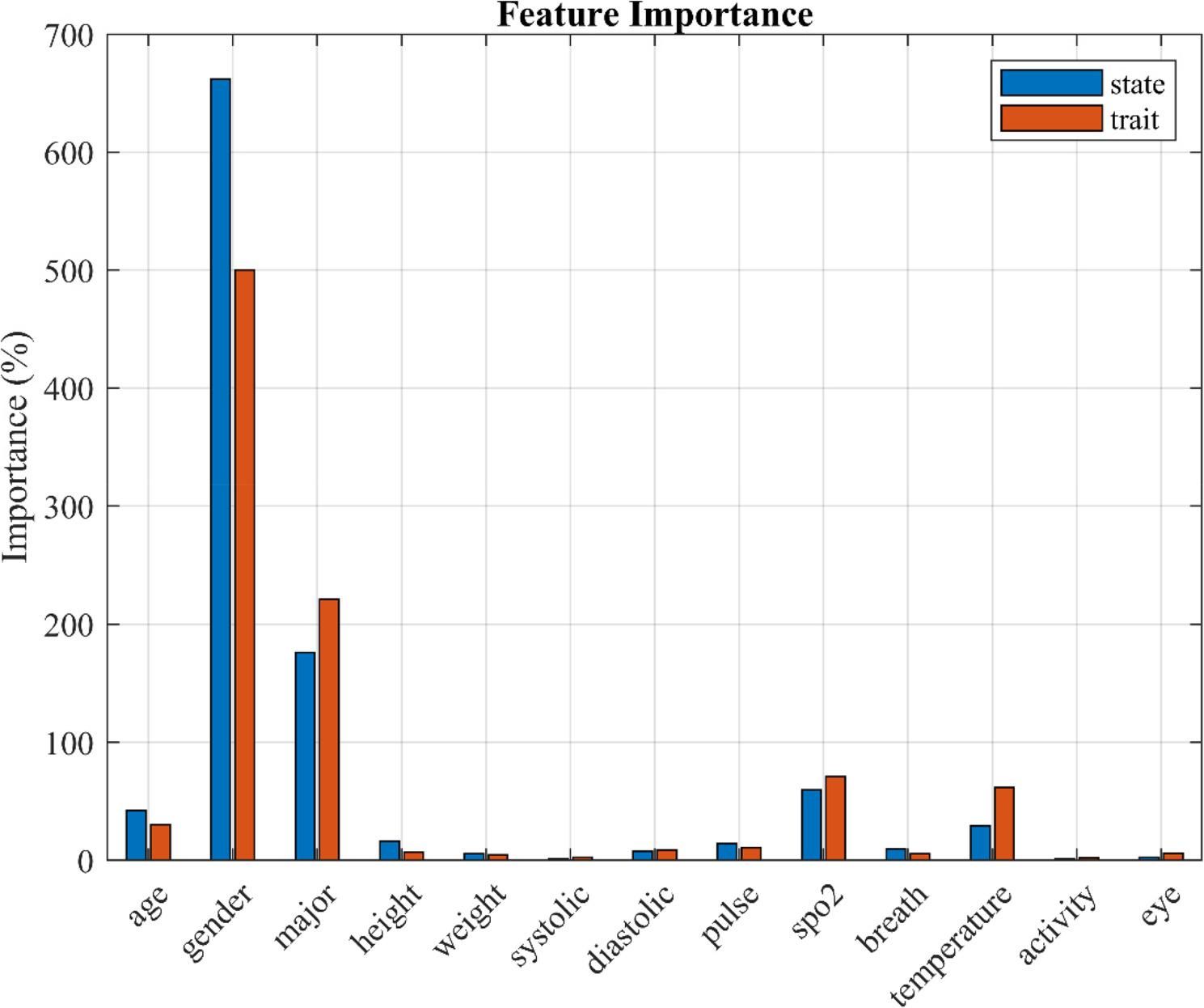
Relative importance of predictors in the multiple linear regression model. This chart shows the relative importance of demographic and physiological features in predicting state anxiety (blue bars) and trait anxiety (orange bars). Gender was the strongest predictor for both anxiety types, while academic major, temperature, and SpO₂ also made substantial contributions. Blood pressure variables and physical activity showed the least influence. Differences in the importance patterns suggest that factors influencing temporary (state) and chronic (trait) anxiety are partially distinct

Figure 2 displays the relative importance of predictors based on standardized regression coefficients, normalized to a percentage scale for comparability. For both state anxiety (blue bars) and trait anxiety (orange bars), gender showed the strongest relative association with anxiety. Academic major, temperature, and SpO₂ also demonstrated notable associations, whereas weight, blood pressure, physical activity, and respiratory rate had minimal contributions. These findings represent relative associations rather than absolute or causal effects and highlight which variables are most strongly related to anxiety levels in this sample. Differences in importance patterns suggest that the factors associated with temporary (state) and chronic (trait) anxiety are partially distinct.

## Discussion

This study utilized a multiple linear regression framework to explore potential associations among demographic and physiological variables and anxiety levels among nursing students. Rather than aiming for individual-level diagnostic precision, the analysis sought to identify system-level patterns of association and evaluate the role of machine learning as an exploratory, complementary tool in nursing education research.

Consistent with previous studies, most demographic and physiological variables were not independently associated with state or trait anxiety [14]. A significant correlation between state and trait anxiety was observed (*p* < 0.001), aligning with the STAI theoretical framework [15]. The model captured general trends but performed moderately at extreme values—a known characteristic of linear regression—suggesting that these findings should be viewed as aggregate indicators rather than individual predictors.

Interpretation of Variables The relationship between marital status and anxiety must be interpreted strictly as a contextual association rather than a causal link. Marital status may function as a proxy for broader life circumstances, such as domestic or financial responsibilities, which correlate with stress levels. Within a predictive framework, such variables serve as indicators for identifying cohorts that may benefit from additional institutional support, without implying direct causation.

Machine learning analysis indicated that gender, academic major, SpO2, and age were among the influential variables within this specific dataset. These results demonstrate that ML can reveal multivariate patterns that are not always evident in traditional testing [16, 17]. However, these findings are exploratory and hypothesis-generating, highlighting relative associations within the model rather than causative effects.

The vulnerability of nursing students to anxiety, exacerbated by intense clinical rotations and demanding academic workloads, mirrors findings in broader medical student populations, where intense pressures necessitate the integration of proactive screening tools [18]. Aligning with the frameworks of Ujkani et al. [19] and Nti and Ramanayake [20], our study suggests that machine learning can function as a supportive and interpretable indicator [19, 20], providing insights that are most effectively utilized at the cohort level to inform institutional planning. By providing explainable insights (XAI), such models enable nursing faculties to transition toward proactive institutional planning—such as the strategic allocation of counseling resources or adjusting clinical schedules—rather than relying on reactive individual measures. Importantly, treating predictors as general learner traits rather than as causative influences facilitates a more supportive, evidence-based environment. By leveraging these insights, nursing faculties can move toward personalized education tailored to individual psychological needs [21, 22]. provided these tools are used as complementary aids rather than absolute diagnostic instruments.

Predicting mental health via machine learning involves complex ethical challenges. As noted by Lawrie et al. [23], applying these algorithms raises questions regarding whether individuals wish to know their risk level and the potential adverse effects of early diagnosis [23]. Furthermore, Ajmani et al. [24] highlight an “Ought-Is” gap—a discrepancy between ideal ethical standards and actual practice—advocating for increased transparency in research disclosures [24]. Accordingly, the present study frames machine learning outputs as voluntary, non-diagnostic, and supportive tools intended to complement—not replace—human judgment and established assessment instruments. Transparent communication and ethical data governance are essential to ensure that such models prioritize student well-being over algorithmic decision-making.

Given the model’s moderate explanatory power, the findings should be interpreted cautiously and used to inform exploratory insights rather than individual-level predictions. Limitations of this study include the modest sample size (*N* = 106), restriction to a single faculty, and the use of bivariate analyses without correction for multiple comparisons, which were applied for exploratory purposes only. Additionally, reliance on a single linear regression model may limit the capture of nonlinear relationships. Future research should examine larger, multi-institutional datasets and consider ensemble or nonlinear machine learning approaches while maintaining interpretability and ethical transparency.

## Conclusion

This study confirms the strong reciprocal relationship between state and trait anxiety and demonstrates that, in this specific population, social and contextual factors—including gender, academic major, and marital status—exert a greater influence on anxiety levels than physiological indicators. The findings also highlight the utility of machine learning models in identifying complex patterns and relative associations that may not be evident through traditional statistical analyses. These results support the potential application of such models for more nuanced assessment, prediction, and targeted intervention strategies to manage anxiety among medical students.

## Supplementary Information

Supplementary Material 1.

## Data Availability

The data supporting the findings of this study are available upon reasonable request from the corresponding author. The materials used in the study, including the educational content and assessment tools, can also be provided upon request.

## Abbreviations

HR: Heart Rate
BP: Blood Pressure
SpO₂: Oxygen Saturation
PA: Physical Activity
ES: Eye Score
ML: Machine Learning
AI: Artificial Intelligence
R²: Coefficient of Determination
RMSE: Root Mean Square Error
MAE: Mean Absolute Error
ANOVA: Analysis of Variance
SD: Standard Deviation
