# Supplementary material for: Exploratory analysis of machine learning models for state and trait anxiety based on Spielberger questionnaire data in nursing students
Source: BMC Med Educ. 2026 Feb 21;26:507. doi: 10.1186/s12909-026-08842-3 (PMC13032597; doi:10.1186/s12909-026-08842-3)
Supplement: Supplementary file 1 — Supplementary Material 1. [file 12909_2026_8842_MOESM1_ESM.docx]

clc

clear

close all

X = xlsread('data.xlsx',1,'A4:N109');

Y = xlsread('result.xlsx',1,'B2:B107');

X(:,12) = [];

varNames = {'age','gender','major','height','weight','systolic', ...

'diastolic','pulse','spo2','breath','temperature','activity','eye'};

tbl = array2table(X,'VariableNames',varNames);

tbl.Result = Y;

tbl.major = categorical(tbl.major);

numVars = setdiff(varNames,{'major'});

for i = 1:length(numVars)

tbl.(numVars{i}) = fillmissing(tbl.(numVars{i}),'median');

end

numTbl = tbl(:,numVars);

numTbl = varfun(@zscore,numTbl);

tblZ = [numTbl tbl(:,{'major','Result'})];

mdl = fitlm(tblZ,'Result ~ age + gender + major + height + weight + systolic + ...

diastolic + pulse + spo2 + breath + temperature + activity + eye');

Y_pred = predict(mdl,tblZ);

RMSE = sqrt(mean((tblZ.Result - Y_pred).^2));

R2 = 1 - sum((tblZ.Result - Y_pred).^2) / ...

sum((tblZ.Result - mean(tblZ.Result)).^2);

disp(RMSE)

disp(R2)

coefVals = abs(mdl.Coefficients.Estimate(2:end));

importance = coefVals ./ max(coefVals) * 100;

figure

bar(importance)

set(gca,'XTickLabel',mdl.CoefficientNames(2:end), ...

'XTickLabelRotation',45)

ylabel('Relative standardized importance (%)')

title('Feature Importance')

grid on

figure

plot(tblZ.Result,'k','LineWidth',1.5)

hold on

plot(Y_pred,'r--','LineWidth',1.5)

xlabel('Sample index')

ylabel('Result')

legend('Observed','Model output')

grid on
